# Fertility-sparing surgery in early-stage cervical cancer: laparoscopic versus abdominal radical trachelectomy

**DOI:** 10.1186/s12905-022-01826-7

**Published:** 2022-06-18

**Authors:** Zuoxi He, Ce Bian, Chuan Xie

**Affiliations:** 1 Department of Gynecology and Obstetrics, West China Second University Hospital, Sichuan University, Chengdu, Sichuan Province People’s Republic of China; 2 Key Laboratory of Birth Defects and Related Diseases of Women and Children, Ministry of Education, Chengdu, Sichuan Province People’s Republic of China

**Keywords:** Radical trachelectomy, Cervical cancer, Fertility-sparing surgery, Pregnancy

## Abstract

**Background:**

Radical trachelectomy is an acceptable alternative to radical hysterectomy for patients with early-stage cervical cancer who wish to preserve reproductive function. This study is designed to compare the laparoscopic versus abdominal radical trachelectomy and provide oncological and obstetric outcome data on patients who have undergone fertility-sparing surgery.

**Methods:**

We retrospectively analyzed all early-stage cervical cancer patients who underwent abdominal radical trachelectomy (ART) or laparoscopic radical trachelectomy (LRT) between January 2005 and June 2017 in West China Second University Hospital, Sichuan University. Patients' clinical details and follow-up were obtained from hospital records.

**Results:**

A total of 33 patients (5 with IA1, 2 with IA2, and 26 with 1B1) were included, including 18 patients treated with ART and 15 patients treated with LRT. The median age at initial diagnosis was 30.00 ± 4.30 years (range 22–39). The mean follow-up time was 74.67 months. Among the 33 patients, 2 patients (6.06%, 1 abdominal/1 laparoscopic) developed recurrence, and there are no evidence of disease for the remaining 31 patients till now. The overall survival rate 96.99% (32/33). The LRT group had a shorter hospital stay (P = 0.01) and less blood loss (P < 0.01) than the ART group. There is no significant difference in the length of operative time (P = 0.48) between the two surgical routes. Overall, 15/33 patients (45.45%) have tried to conceive. 6 (40.00%) patients were pregnant and 6 (40.00%) patients were infertility. The ART group had a higher clinical pregnancy rate (P = 0.03) than the LRT group.

**Conclusions:**

There is no statistically significant difference in oncological outcome between the two surgical approaches. The clinical pregnancy rate in the ART group was significant higher than that in the LRT group. However, LRT resulted in less blood loss and decreased length of hospital stay.

## Background

Cervical cancer ranks fourth among the most commonly diagnosed cancers in women worldwide, and it ranks second in incidence and mortality in lower human development index areas [1]. According to the cancer statistics from the U.S. Cancer Statistics Working Group, the incidence for cervical cancer is reported to be 47.30 per 100,000 women in reproductive-aged women (20–45 years) [2]. In China, there were 98.9 thousand women newly diagnosed with cervical cancer each year, with 29.7 thousand under the age of 45 [3]. With the younger onset age of cervical cancer and the postponement of the childbearing age of women [4, 5], the fertility-sparing surgery will become an alternative method for more patients.

The fertility-sparing surgery include conization, simple trachelectomy and radical trachelectomy (RT). The 2020 NCCN guidelines recommend conization for patients in IA1 without lymphatic vascular invasion (LVSI), RT suggested for patients in IA2 ~ IB1 and selective IB2 [6]. It has been reported that conization combined with neoadjuvant chemotherapy (NACT) is used in IB1 patients without medium and high risk [7, 8]. Morice P et al. [9] found that the oncological results were remarkably similar in patients with stage IB1 cevical cancer treated by different surgical modalities (conization, simple trachelectomy and RT). Fertility-sparing surgery could be performed after NACT for early-stage cervical cancer patients with tumor diameter of 2–4 cm who require fertility preservation [10]. In addition, Viveros-Carreno D et al. [11] reported the fertility-sparing surgery combined with NACT in early-stage cervical cancer women with tumor larger than 4 cm in diameter, and the 4.5-year disease-free survival was 92.3% and the 4.5-year overall survival rate was 100%.

The surgical routes of RT include transabdominal, minimally invasive and transvaginal. Scope of RT usually preserve the uterine body, cut off the cervix at 5 mm under the uterine isthmus and cut off 1–2 cm vagina and a certain range of para-uterine tissue. Meanwhile, cerclage of cervix can be performed during the fertility-sparing surgery. At present, the main surgical modalities of RT are laparoscopic, transvaginal and transabdominal. A systematic review [12] summarize the number of patients intending to conceive and the actual number of pregnancy in 47 studies and found that the average clinical pregnancy rate after radical trachelectomy was 53.6%, and the vaginal radical trachelectomy (VRT) had a higher mean clinical pregnancy rate than abdominal radical trachelectomy (ART). Additionally, a recent systematic review at 2020 [13] reported the median recurrence rates were 3.8%, 3.3%, 0% for vaginal, abdominal and laparoscopic radical trachelectomy (LRT) respectively.

Differing surgical modalities of RT may result in disparate oncologic and obstetric outcomes, as evidenced by the multi-center randomized laparoscopic approach to cervical cancer trial [14]. Data on direct comparisons of abdominal and laparoscopic trachelectomy in the terms of oncologic and obstetric outcomes are lacking. The current work aimed to evaluate the long-term results of abdominal and laparoscopic trachelectomy performed in early-stage cervical cancers at a university hospital setting, where primary cervical cancer care is centralized in western China. Meanwhile, the direct comparisons of abdominal and laparoscopic trachelectomy in the terms of surgical, oncologic, fertility and obstetric outcomes was also performed in this study.

## Methods

This study was reviewed and approved by the ethics committee and the data inspectorate of West China Second University Hospital of Sichuan University and the methods were carried out in accordance with the relevant guidelines and regulations. Informed consent was obtained from all participants.

The inclusion criteria were as follows: (1) young age (< 40 years old); (2) clinical stage IA1 to IB2 (2018 FIGO staging system [15]) and tumor size ≤ 2 cm; (3) received LRT or ART surgery; (4) diagnosed from January 2005 to June 2017 in West China Second University Hospital of Sichuan University; (5) Cervical cancer diagnoses were confirmed through pathological examination of a cervical biopsy; (6) At least two experienced gynecologic oncologists were involved in determining the clinical stage. The exclusion criteria included (1) age ≧ 40 years; (2) previous subtotal hysterectomy history; (3) received simple or radical hysterectomy less than 1 year after RT.

A total of 34 patients with early cervical cancer were collected. The data extracted from the medical records include the age at diagnosis, clinical stage, histological type, surgical route, operative time, estimated blood loss, use of cerclage, and length of hospital stay. The pathological data included residual tumor, positive surgical margin, para-uterine involvement, lymphatic vascular invasion and lymph node status. Follow-up contents include: postoperative complications (amenorrhea, dysuria, lymphoid cyst, abdominal pain), trying-to-conceive or not, the length of trying-to-conceive time, pregnancy or not. (i) For the pregnant patient, the follow-up contents including the pregnancy mode, gestational week at the time of delivery, delivery mode, pregnancy complications; (ii) For patients unable to conceive, the follow-up contents including the length of infertility and the cause of infertility. The clinical pregnancy rate is defined as the number of patients with pregnancy out of the total number of patients who attempted to conceive.

Data were analyzed using SPSS 25.0 software. All quantitative data were presented as mean ± SD or median (range). An independent t-test was used to compare the differences between the two groups. Further, classified variables were analyzed using the chi-square (χ2) test or the rank-sum test. A P-value < 0.05 was considered statistically significant.

## Results

### Patients and basic characteristics

There were 34 patients with early-stage cervical cancer who underwent LRT or ART in our hospital from January 2005 to June 2017. Among them, one was excluded because the postoperative pathological examination of this patient indicated the metastasis of right pelvic lymph nodes, and she received supplementary radical hysterectomy one month after the LRT. Therefore, a total of 33 patients were included in this study, including 18 patients treated with ART and 15 patients treated with LRT. The basic characteristics of the 33 patients are summarized and listed in Table 1. The average age at initial diagnosis was 30.00 ± 4.30 years (range 22–39). Among the 33 patients, most of the patients attempted to conceive, and the rest of them insisted to preserve the uterus and menstrual patterns even though they did not have a strong willingness to conceive. For those patients who just preserve the uterus and menstrual patterns but not to conceive, we mainly follow-up the oncology outcome of them. Meanwhile, some patients’ trying-to-conceive time are less than one year, it is not allowed to define them as infertility.

**Table 1:** Basic characteristics of patients with early-stage cervical cancer who underwent ART or LRT

	Total n = 33	NED n = 31	Recurrence n = 2
Age at surgery, mean ± SD	30.00 ± 4.30	29.97 ± 4.42	30.50 ± 2.12
*FIGO stage 2018 (n)*			
IA1	3	3	0
IA1 + LVSI	2	2	0
IA2	2	2	0
IB1	26	24	2
*Histological type (n)*			
SCC	24	22	2
AC	6	6	0
ASC	3	3	0
NACT (n)	4	3	1
Postoperative chemotherapy (n)	4	3	1
Pelvic lymph node(s) metastasis (n)	0	0	0
*Surgical modality (n)*			
LRT (n)	15	14	1
ART (n)	18	17	1
Trying-to-conceive (n)	15	15	0
Pregnancy (n)	6	6	0
Infertility (n)	6	6	0
Follow up (months), median (range)	60.00 (7,180)	60.00 (7,176)	84.50 (24,145)
Death (n)	1	0	1

*AC* adenocarcinoma, *ART* abdominal radical trachelectomy, *ASC* adenosquamous carcinoma, *LRT* laparoscopic radical trachelectomy, *LVSI* lymph vascular space invasion, *NED* no evidence of disease, *SCC* squamous cell carcinoma, *VRT* vaginal radical trachelectomy

Of the 33 patients, most of the patients were in IB1 stage, accounting for 78.79%. 5 patients (15.15%) had stage IA1 tumors, 2 patients (6.06%) had stage IA2 tumors. Among the 5 patients with stage IA1 tumors, 2 patients had lymph vascular space invasion (LVSI) by postoperative pathologic examination. 3 patients who were diagnosed with stage IA1 without LVSI were submitted first to conization and later to RT because of the positive margin after conization. The average age of these patients at diagnosis was 30.00 ± 4.30 (range 22–39). As for the tumor pathological classification, most of the patients were diagnosed with the squamous cell carcinoma (SCC), accounted for 72.73%. 6 (18.18%) patients had adenocarcinoma (AC), and 3 (9.09%) patients had adenosquamous carcinoma (ASC). Among the 33 patients who received RT, 18 patients (54.55%) underwent ART and 15 patients (45.45%) underwent LRT. Except for the two IA1 patients, the remaining 31 patients underwent lymphadenectomy. Among the 33 patients, 4 patients received NACT and 4 patients received postoperative chemotherapy. 4 patients received NACT because of their serious complications that needed to be treated before the operation. 1 patient received postoperative chemotherapy due to parametrial lymph node metastasis. 2 patients received postoperative chemotherapy due to deep invasion of cervical stroma, and the fourth patient relapsed two years after the laparoscopic radical trachelectomy, and then she received radical hysterectomy and postoperative concomitant chemo-radiation. None of the 33 patients was treated with radiation therapy at the time of initial treatment. Of the 33 patients, there were 15 (45.45%) patients tried to conceive. Of the 15 patients attempted to conceive, 6 (40.00%) patients were pregnant and 6 (40.00%) patients were infertility. The rest 3 patients prepare for pregnancy less than one year.

### Oncological outcome

The mean follow-up time was 74.67 ± 55.73 months. Among the 33 patients, 2 patients (6.06%) developed recurrence, and there are no evidence of disease for the remaining 31 patients till now. One patient died of recurrence, the overall survival rate 96.99% (32/33). Of the two patients who recurred, one patient experienced recurrence 10 years after the surgery. This patient had stage IB1 cervical squamous cell carcinoma at the time of initial diagnosis. She received ART and 2 cycles preoperative combination chemotherapy of platinum and paclitaxel. None of postoperative chemotherapy and radiation therapy was performed. This patient had been trying to conceive but never get pregnant. She was diagnosed with recurrent cervical cancer with distant metastasis and died before receiving salvage treatment 1 years after the recurrence. The another patient who relapsed was treated with LRT and experienced recurrence 2 years after the initial surgery, and then she underwent radical hysterectomy followed by 4 cycles postoperative combination chemotherapy of platinum and paclitaxel. There is no evidence of disease after the second surgery up to now. The postoperative pathological examination indicated that there was one patient with para-uterine lymph node metastasis. This patient should be treated with a concomitant chemo-radiation, but she refused radiotherapy and only received chemotherapy because of her strong desire to preserve fertility. However, this patient showed no evidence of disease after follow-up. Additionally, there were no intra-operative complications. The decreased menstrual is the main complication among these patients and there were no postoperative complications (such as amenorrhea, dysuria, lymphoid cyst and abdominal pain) among the 33 patients.

### The comparation of ART group and LRT group

The comparation of the ART group and LRT group is listed in Table 2. We compared the operative outcomes of the 2 groups. There is no significant difference in the length of operative time (P = 0.48) between the two surgical modalities As expected, The LRT group had a shorter hospital stay (P < 0.05) and less estimated blood loss (P < 0.05) than the ART group, while the clinical pregnancy rate in the ART group was significant higher than that in the LRT group (P = 0.03). Because ART was started earlier than LRT in our institute, ART group has a longer postoperative follow-up time than LRT group (P < 0.01). Furthermore, there is no significant difference in recurrence rate (P = 1.00) and survival rate (P = 1.00) between the two group. And the total recurrence rate is 6.06%. Additionally, postoperative pathological examination indicated that there is no patient with positive surgery margins and residual tumor in both groups.

**Table 2:** The comparation between the two surgical approaches

	ART (n = 18)	LRT (n = 15)	P value
*FIGO stage 2018 (n)*			
IA1	1	2	0.38
IA1 + LVSI	2	0	
IA2	2	0	
IB1	13	13	
Estimated blood loss (ml, M ± SD)	716.67 ± 308.07	232.00 ± 149.77	< 0.05
Hospital stay (days, M ± SD)	8.50 ± 2.36	6.20 ± 2.34	< 0.05
Operative time(mins, M ± SD)	299.44 ± 88.05	316.00 ± 39.06	0.48
Pregnancy/trying-to-conceive(n/n)	3/6	3/9	0.03
Clinical pregnancy rate	50.00%	33.33%	0.03
Follow up (months, M ± SD)	113.56 ± 46.12	28.00 ± 15.45	< 0.01
Recurrence (n)	1	1	1.00
Death (n)	1	0	1.00

### Fertility and obstetric outcomes

Overall, 15/33 (45.45%) patients have tried to conceive. 6 (40.00%) patients were pregnant and 6 (40.00%) patients were infertility. The remaining 3 patients prepare for pregnancy less than one year. We collected the clinical data of the six pregnant patients and listed in Table 3. Two of them are in pregnancy at present and four of them have delivered five times in total, including two premature labour (one due to placental abruption, the another one due to regular uterine contraction) and three full-term deliveries. All the infants were born alive and with a good outcome after follow-up. 2 (33.33%) of the pregnant patients underwent cerclage during the cervical surgery. Of the seven pregnancies, 2 (28.57%) were from assisted reproduction and the rest 5 (71.43%) were from natural conception. All the pregnancies occurred at the mean interval 3.14 years after the surgery. Furthermore, all those patients delivered by cesarean section and has a good oncological and obstetric outcomes.

**Table 3:** Characteristics of patients with successful pregnancy

No	SG	stage	Surgery route	GA at delivery (w)	Causes of termination	Obstetrical complication	Conception mode	Interval (y)	Newborn outcome
1	1	IA1	ART	39	Term delivery	N/A	IVF	5	Good
2	1	IB1	LRT	29	PA	PA	NC	1	Good
3	1	IB1	ART	23w at present	N/A	N/A	IVF	3	Good
4	1	IB1	LRT	15w at present	N/A	N/A	NC	2	Good
5	2	IB1	ART	37 + 4	Term delivery	N/A	NC	3	Good
				37	Term delivery	N/A	NC	5	Good
6	1	IB1	LRT	34 + 2	TPL	TPL	NC	3	Good

*GA* gestational age, *N/A* not applicable, *NC* natural conception, *PA* placental abruption, *SG* subsequent gravida, *TPL* threatened preterm labor

### Infertility after surgery

Among the 33 patients who underwent RT, there were 15 patients who attempted to get pregnant. Of the 15 patients, 6 patients were diagnosed with infertility. As for the causes of infertility, there was one patient (1/6) with cervical stenosis, three patients (3/6) with fallopian tube obstruction, one patient with ovulation disorder (1/6) and the remaining patient with other unknown factor (without systematic infertility examination). Among known causes for infertility, ovulation disorder, cervical stenosis and fallopian tube obstruction were the most frequent ones in patients after RT. Of the 6 patients with failure of pregnancy, one patient had infertility before surgery.

## Discussion

Due to the younger onset of cervical cancer and late marriage of contemporary women, many patients with early cervical cancer have not completed childbirth. Therefore, more attention is paid to fertility-sparing surgery. RT has been developed as a fertility-sparing surgery for early-stage cervical cancer. RT was firstly performed by Dargent via vaginal route in 1986 [16], but commonly referenced of publication was in 1994. Correspondingly, the ART and LRT were reported in 1997 and 2005 respectively. Different surgical modalities for the treatment of cervical cancer, however, may lead to diverse oncologic results, as evidenced by the multi-center randomized Laparoscopic Approach to Cervical Cancer trial [14]. Although the Laparoscopic Approach to Cervical Cancer trial addresses radical hysterectomy, these data provoke the question of whether its results can be extrapolated to any surgical modalities for cervical cancer. Hence, we performed a retrospective review of 33 cases of early-stage cervical cancer diagnosed and treated at a single institution from January 2005 to June 2017 and investigated the oncological and obstetric outcomes of the abdominal and laparoscopic RT. In this study, there is no significant difference in postoperative recurrence rate and overall survival rate between the two surgical modalities. However, the ART group had a higher clinical pregnancy rate than LRT group. The total recurrence rate in our study is 6.06% (2/31), which is higher than the median recurrence rate 3.3% reported in a systematic review [13]. We consider this to be acceptable in a retrospective cohort. Furthermore, it is thought to be oncological equal to a radical hysterectomy when the recurrence rates is between 1.8 and 7.0% [17].

The total clinical pregnancy rate in our study is 40% (6/15), which is comparable to that published in a previous research [18]. However, the clinical pregnancy rate is conspicuously lower than that in reported patients who underwent VRT, ART or LRT alone [12, 17, 19, 20]. We consider that the lower clinical pregnancy rate may be related to the shorter follow-up time of patients who underwent LRT (of the 9 non-pregnancy patients in our study, 4 patients underwent surgery after 2018, and all the 4 patients underwent LRT). As we know, the clinical pregnancy rate after RT is affected by many factors. The cervical factors, including cervical stenosis, the length of cervix and the absent cervical mucous, were considered to be the most important causes for infertility in patients treated with RT [17, 21, 22]. It is widely considered that patients with cervical length less than 1 cm are more likely to be infertility than patients with cervical length ≥ 1 cm [17]. In this study, there was one patient (1/6) with cervical stenosis, three patients (3/6) with fallopian tube obstruction, one patient with ovulation disorder (1/6) and the remaining patient with other unknown factor (this patient did not undergo systematic infertility examination).

The LRT offered a number of advantages such as improved visualization, less blood loss, and faster recovery in our study, which was also proved by previous studies [18, 23, 24]. In our study, there was no significant difference in operative time and histopathologic outcomes between the two surgical approaches. However, the operative time of LRT is reported to be longer when compared to that of ART in other study [23]. As laparoscopic radical trachelectomy requires refined skills, the laparoscopic approach may increase the difficulty of the operation and increase the operation time when it is not performed skillfully. As for the fertility outcome of the two surgical modalities, the higher pregnancy rate was observed in ART group when compared with the LRT group [13, 18]. Nevertheless, the pregnancy rate of the robot-assisted ART can reach to 81% [17], which is comparable to that of VRT. Hence, the relatively lower pregnancy rate in LRT group may be related to the shorter follow-up time.

The total recurrence rate of RT is at a low level. In previous studies, the median recurrence rate was reported to be 3.3% (range 0–25%) after a median follow-up of 48 months (range 2–202 months) [13]. In most of studies on LRT, the recurrent rate was 0%—4% in each reported article [17, 18, 20, 23, 24], which may be related to the short follow-up time. Park J Y et al. [25] reported 9 (9/79) recurrent cases after a median follow-up time of 44 months in patients treated with LRT and concluded that the tumor size greater than 2 cm and a depth of stromal invasion greater than 50% were the main risk factors for recurrence. In this study, 2 patients (1 abdominal/1 laparoscopic)) developed recurrence, and the the total recurrence rate of RT was 6.06%. Additionally, there is no significant difference in postoperative recurrence rate and overall survival rate between the two surgical modalities. However, Ramirez et al. [14] reported a multicenter, prospective randomized controlled study of minimally invasive manual surgery for cervical cancer, and pointed out that minimally invasive treatment of cervical cancer has lower disease-free survival and overall survival when compared with laparotomy. Nevertheless, this study has limitations. The minimally invasive arm was heavily weighted toward laparoscopic surgery in this study, which may not reflect the current practices. Furthermore, it was a multinational and multicenter study with different surgical skills. Therefore, further studies are needed to determine whether minimally invasive surgery affects oncological outcomes in patients with early-stage cervical cancer. Although, long-term benefits of performing laparoscopic fertility-sparing radical trachelectomy remain to be delineated, women with early-stage cervical cancer could be offered a minimally invasive surgical modality when undergoing radical trachelectomy.

RT has been reported to be a feasible treatment for patients with tumors ≦ 2 cm in most studies. However, Fertility-sparing surgery has been controversial for early-stage cervical cancer patients with tumors larger than 2 cm. NACT could reduce the tumor volume and may effectively inhibit the micrometastases of paracervical tissue and pelvic lymph nodes [26, 27]. Therefore, it has been reported that NACT combined with conization is used for early-stage cervical cancer patients with tumors less than 2 cm in diameter [7, 28] and NACT combined with RT surgery for early-stage cervical cancer patients whose tumors larger than 2 cm in diameter [10, 11, 29]. Viveros-Carreno D et al. [11] even reported the fertility-sparing surgery combined with NACT was used in early-stage cervical cancer women with tumors larger than 4 cm in diameter, and the 4.5-year disease-free survival was 92.3% and the 4.5-year overall survival rate was 100%. In our study, there were four patients who received NACT, and the reason why the four patients received NACT was that these patients required delayed surgery because of the serious complications that needed to be treated before the operation.

The limitations of this study lie within its retrospective nature, the small number of patients and the lack of randomization. In addition, four patients received NACT due to delayed surgery and one patient with parauterine lymph node metastasis received only postoperative chemotherapy, which may lead to the bias of this study. Furthermore, a longer follow-up is needed to further evaluate the clinical pregnancy rate and oncological outcome. Nonetheless, we believe this study should be useful for patients with early-stage cervical cancer as well as gynecologists considering RT.

## Conclusions

In summary, despite a small cohort of patients, this study suggests the feasibility of laparoscopic fertility-sparing radical trachelectomy in appropriately selected women with early-stage cervical cancer. In keeping with findings in the current literature, our study supports the conclusion that the total recurrence rate of RT is at a low level. The total clinical pregnancy rate in this study is 40% (6/15), which is also comparable to that published in current literature. The laparoscopic surgery approach resulted in less blood loss and decreased length of postoperative hospital stay when compared to the laparotomy approach. Furthermore, there was no difference in histopathologic outcomes and operative time among patients undergoing laparoscopic radical trachelectomy when compared to laparotomy. Additionally, there was no statistically significant difference in oncological outcomes between the two surgical modalities, The clinical pregnancy rate in the ART group was significant higher than that in the LRT group, however, the lower clinical pregnancy rate in the LRT group may be related to the shorter follow-up time of patients who underwent LRT. Base on these findings, this study could supports the safety of RT via a laparoscopic surgery approach. This study is only a retrospective analysis in a single institution but could be helpful for patients with early-stage cervical cancer as well as gynecologists considering RT. In the future, a multi-center large-sample randomized controlled clinical trial is needed to evaluate the role of fertility-sparing techniques in young patients with early-stage cervical cancer.

## Data Availability

The datasets used and/or analysed during the current study available from the corresponding author on reasonable request.

## Abbreviations

RT: Radical trachelectomy
ART: Abdominal radical trachelectomy
LRT: Laparoscopic radical trachelectomy
VRT: Vaginal radical trachelectomy

## References

[1] Bray F, Ferlay J, Soerjomataram I (2018). Global cancer statistics 2018: GLOBOCAN estimates of incidence and mortality worldwide for 36 cancers in 185 countries. CA Cancer J Clin.

[2] U.S. Cancer Statistics Working Group. U.S. Cancer Statistics: Data Visualizations Tool. Based on November 2018 submission data (1999–2016). Atlanta, GA: U.S. Department of Health and Human Services, Centers for Disease Control and Prevention and National Cancer Institute.

[3] Chen W, Zheng R, Baade PD (2016). Cancer statistics in China, 2015. CA Cancer J Clin.

[4] Liu P (2018). Big data evaluation of cervical cancer clinical epidemiology in mainland china for 13 years. Chin J Pract Gynecol Obstetr.

[5] Cheng ZW, Yang YL, Jia XC (2018). Analysis of Chinese population fertility trend based on age-period-cohort model. J Zhengzhou Univ Med Sci.

[6] Abu-Rustum NR, Yashar CM, Bean S (2020). NCCN guidelines insights: cervical cancer, version 1.2020. J Natl Compr Canc Netw.

[7] Tomao F, Corrado G, Peccatori FA (2016). Fertility-sparing options in young women with cervical cancer. Curr Treat Options Oncol.

[8] Tomao F, Maruccio M, Preti EP (2017). Conization in early stage cervical cancer: pattern of recurrence in a 10-year single-institution experience. Int J Gynecol Cancer.

[9] Morice P, Maulard A, Scherier S, Sanson C (2022). Oncologic results of fertility sparing surgery of cervical cancer: an updated systematic review. Gynecol Oncol.

[10] Plante M, van Trommel N, Lheureux S (2019). FIGO 2018 stage IB2 (2-4 cm) cervical cancer treated with neo-adjuvant chemotherapy followed by fertility sparing surgery (CONTESSA); neo-adjuvant chemotherapy and conservative surgery in cervical cancer to preserve fertility (NEOCON-F). A PMHC, DGOG, GCIG/CCRN and multicenter study. Int J Gynecol Cancer.

[11] Viveros-Carreno D, Rodriguez J, Rendon Pereira GJ (2022). Fertility-sparing surgery after neo-adjuvant chemotherapy in women with cervical cancer larger than 4 cm: a systematic review. Int J Gynecol Cancer.

[12] Nezhat C, Roman RA, Rambhatla A (2020). Reproductive and oncologic outcomes after fertility-sparing surgery for early stage cervical cancer: a systematic review. Fertil Steril.

[13] Smith ES, Moon AS, O'Hanlon R (2020). Radical trachelectomy for the treatment of early-stage cervical cancer: A systematic review. Obstet Gynecol.

[14] Ramirez PT, Frumovitz M, Pareja R (2018). Minimally invasive versus abdominal radical hysterectomy for cervical cancer. N Engl J Med.

[15] Bhatla N, Aoki D, Sharma DN (2018). Cancer of the cervix uteri. Int J Gynecol Obstet.

[16] Dargent D, Martin X, Sacchetoni A (2000). Laparoscopic vaginal radical trachelectomy: a treatment to preserve the fertility of cervical carcinoma patients. Cancer.

[17] Johansen G, Lonnerfors C, Falconer H (2016). Reproductive and oncologic outcome following robot-assisted laparoscopic radical trachelectomy for early stage cervical cancer. Gynecol Oncol.

[18] Vieira MA, Rendon GJ, Munsell M (2015). Radical trachelectomy in early-stage cervical cancer: a comparison of laparotomy and minimally invasive surgery. Gynecol Oncol.

[19] Zusterzeel PL, Pol FJ, van Ham M (2016). Vaginal radical trachelectomy for early-stage cervical cancer: increased recurrence risk for adenocarcinoma. Int J Gynecol Cancer.

[20] Ebisawa K, Takano M, Fukuda M (2013). Obstetric outcomes of patients undergoing total laparoscopic radical trachelectomy for early stage cervical cancer. Gynecol Oncol.

[21] Li J, Li Z, Wang H (2011). Radical abdominal trachelectomy for cervical malignancies: surgical, oncological and fertility outcomes in 62 patients. Gynecol Oncol.

[22] Boss EA, van Golde RJ, Beerendonk CC (2005). Pregnancy after radical trachelectomy: a real option?. Gynecol Oncol.

[23] Kucukmetin A, Biliatis I, Ratnavelu N (2014). Laparoscopic radical trachelectomy is an alternative to laparotomy with improved perioperative outcomes in patients with early-stage cervical cancer. Int J Gynecol Cancer.

[24] Nick AM, Frumovitz MM, Soliman PT (2012). Fertility sparing surgery for treatment of early-stage cervical cancer: open vs robotic radical trachelectomy. Gynecol Oncol.

[25] Park JY, Joo WD, Chang SJ (2014). Long-term outcomes after fertility-sparing laparoscopic radical trachelectomy in young women with early-stage cervical cancer: an asan gynecologic cancer group (AGCG) study. J Surg Oncol.

[26] Lu Q, Zhang Y, Wang S, Guo S (2014). Neoadjuvant intra-arterial chemotherapy followed by total laparoscopic radical trachelectomy in stage IB1 cervical cancer. Fertil Steril.

[27] Maneo A, Chiari S, Bonazzi C (2008). Neoadjuvant chemotherapy and conservative surgery for stage IB1 cervical cancer. Gynecol Oncol.

[28] Tsaousidis C, Kraemer B, Kommoss S (2022). Large conization-retrospective monocentric results for fertility preservation in young women with early stage cervical cancer. Reprod Sci.

[29] Buda A, Borghese M, Puppo A (2022). Neoadjuvant chemotherapy prior fertility-sparing surgery in women with FIGO 2018 stage IB2 cervical cancer: a systematic review. Cancers.

